# Progress and trends of research on mineral elements for depression

**DOI:** 10.1016/j.heliyon.2024.e35469

**Published:** 2024-07-31

**Authors:** Biao Gao, Chenqi Li, Yicui Qu, Mengyu Cai, Qicheng Zhou, Yinyin Zhang, Hongtao Lu, Yuxiao Tang, Hongxia Li, Hui Shen

**Affiliations:** a Department of Naval Nutrition and Food Hygiene, Naval Medical University, Shanghai, 200433, China; b Teaching and Research Support Center, Naval Medical University, Shanghai, 200433, China; c Department of Nutrition, The Third Affiliated Hospital of Naval Medical University, Shanghai, 200438, China

**Keywords:** Mineral elements, Depression, Status quo, Bibliometric analyses, CiteSpace

## Abstract

**Objective:**

To explore the research progress and trends on mineral elements and depression.

**Methods:**

After querying the MeSH database and referring to the search rules, the search terms were selected and optimized to obtain the target literature collection. We analyzed the general characteristics of the literature, conducted network clustering and co-occurrence analysis, and carried out a narrative review of crucial literature.

**Results:**

Bipolar disorder was a dominant topic in the retrieved literature, which saw a significant increase in 2010 and 2019–2020. Most studies focused on mineral elements, including lithium, calcium, magnesium, zinc, and copper. The majority of journals and disciplines were in the fields of psychiatry, neuropsychology, neuropharmacology, nutrition, medical informatics, chemistry, and public health. The United States had the highest proportion in terms of paper sources, most-cited articles, high-frequency citations, frontier citations, and high centrality citation. Regarding the influence of academic institutions, the top five were King's College London, the Chinese Academy of Sciences, University of Barcelona, INSERM, and Heidelberg University. Frontier keywords included bipolar disorder, drinking water, (neuro)inflammation, gut microbiota, and systematic analysis. Research on lithium response, magnesium supplementation, and treatment-resistant unipolar depression increased significantly after 2013.

**Conclusion:**

Global adverse events may have indirectly driven the progress in related research. Although the literature from the United States represents an absolute majority, its influence on academic institutions is relatively weaker. Multiple pieces of evidence support the efficacy of lithium in treating bipolar disorder (BD). A series of key discoveries have led to a paradigm shift in research, leading to increasingly detailed studies on the role of magnesium, calcium, zinc, and copper in the treatment of depression. Most studies on mineral elements remain diverse and inconclusive. The potential toxicity and side effects of some elements warrant careful attention.

## 1 Introduction

As life expectancy increases and the population ages, non-fatal diseases account for more of the disease burden, increasing the demand for health services [1]. The years of living with disability (YLD) of depression rose to the third position between 1990 and 2017 [2] and became the second leading cause of disability in 2019 [3,4] and 2021 [5]. Micronutrient deficiencies are also a global health problem [6], leading to metabolic disorders, delayed growth and development, reduced resistance to disease, and even morbidity and death in severe cases.

Depression results from a chemical imbalance in the brain, in which stressors and cytokines work together to affect neuroplasticity, altering glial cell neurotransmission [7]. The dilemmas of traditional treatment include high cost, adverse drug reactions, and poor efficacy [8]; however, multiple approaches and multidisciplinary interventions offer new hope. Among depression risk factors, mineral elements play an essential role, and changes in their levels affect neurodevelopment through neurochemical pathways [9], participate in the occurrence and development of depression [10], and can be used as potential diagnostic and prognostic biomarkers [11].

Correcting the imbalanced homeostasis can enhance the efficacy of antidepressants, reduce their dosage and side effects, and promote mental health [12]. In addition, trace nutrients has been found to be beneficial for the response to inflammation response under high-stress conditions [13]**,** thereby contributing to the promotion of psychological well-being [14].

Elements such as zinc, selenium, manganese, iodine, vanadium, magnesium, lithium, iron, calcium, and chromium can be involved in the pathophysiological processes of depression and anxiety, individually or synergistically, either as enzymatic cofactors [15] or as non-enzymatic antioxidant players that exert different target effects.

Research on mineral elements and depression is unevenly distributed and the theoretical system is complex and is rapidly progressing. Furthermore, there are conflicting conclusions [16,17], widely disparate dose-related effects [[18], [19], [20]], complex synergistic/antagonistic effects [[21], [22], [23]], and paradigm shifts brought about by critical new insights [[24], [25], [26], [27]], illustrating the remarkable development history. The next step can be carried out scientifically and effectively only with a macro-understanding of relevant research and an accurate grasp of critical details. Maintaining a high level of clinical practice requires a thorough familiarity with the latest advances and knowledge in the field [28].

We posit that the research field of mineral elements and depression may have changed as a result of the rapid development of omics research methods and the emergence of new drugs or functions. These changes may have resulted in a shift in research frontiers, focus, and even paradigms. To investigate this hypothesis, we used CiteSpace software, a research tool developed and continuously improved by Dr. Chaomei Chen of Drexel University in the United States [29]. This software can reveal the frontiers, hotspots, and development trends of a specific research field in a practical and easy-to-understand model, generating a "scientific knowledge graph." Our published research has confirmed the effectiveness and value [30,31]. We utilized data from the mainstream and representative WOS SCIE (2007 to present) database to provide CiteSpace with literature citations and conduct the most valuable citation analysis. The results of our study can provide new, unique, and strategic references for peers and offer guidance for the next step of research design.

## 2 Materials and methods

### 2.1 Databases and tools

Medical Subject Headings (MeSH) were queried in the MeSH database to select search terms, and the literature was retrieved from the Web of Science (WoS) Core Collection Database (SCIE, from 2007 to the present). SCIE is the only database that provides the reference data for citation analysis, which is essential and most valuable for this study. CiteSpace (version 5.8 R4), NoteExpress (version 3.5.0.9054), and EXCEL2019 were used for data storage, management, and analysis, with the help of the WoS database online platform and drawing tool.

### 2.2 Data acquisition

The search strategy was designed to ensure completeness. We initially consulted MeSH to identify relevant terms and then utilized common words and a list of major mineral element names with wildcards for the search. However, the redundancy of the search terms may have slightly impacted the accuracy of the results. Although some data cleaning was performed prior to analysis, a thorough verification of each entry was not performed. However, this limitation does not hinder the research results, as irrelevant and insignificant literature clusters can be easily disregarded during the analysis [32,33].

We conducted a comprehensive literature search in the SCIE database. The search strategy included two main components: depression-related terms and trace element-related terms. For depression, we searched the MeSH database and identified 23 relevant terms of medical subjects. We optimized the search using wildcards and included the following terms: depressive, depression, bipolar disorder*, bipolar affective, bipolar mood, bipolar depression, nanic disorder*, manic depressive, affective disorder*, mood disorder*, and melancholia*. These terms were searched in the topic field using the "OR" operator. For trace elements, we focused on chromium (Cr), cobalt (Co), copper (Cu), fluorine (F), iodine (I), iron (Fe), manganese (Mn), molybdenum (Mo), selenium (Se), and zinc (Zn) because they are associated with mental illness. Furthermore, the macroelement lithium (Li) is often used in the treatment of bipolar disorder (BD) [34], and lead, calcium, and magnesium [35,36] are also associated with depression. Therefore, these minerals were included in our study. MeSHs were queried, referring to the English dictionary and search rules to obtain the search terms: cobalt*, chromi*, copper*, fluorid*, iodi*, iron*, acid-iron, mangan*, molybd*, seleni*, zin*, lithium*, calcium*, calpain*, calmodulin*, calreticulin, magnesi*, and lead exposure. These terms were searched in the topic field using the "OR" operator.

**Fig. 1:**
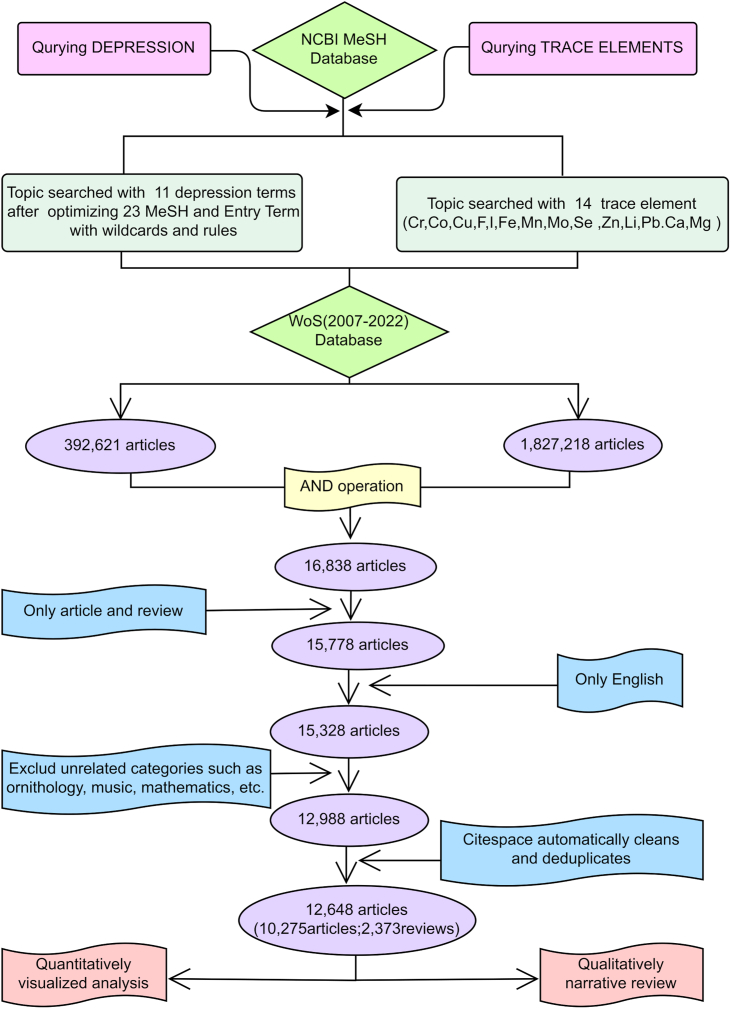
Data acquisition flow chart.

We include articles and reviews published in English, excluding conference papers, books, book chapters, and other types of documents. Articles from irrelevant categories such as ornithology, music, and mathematics were also removed. The search results were imported into CiteSpace for verification and cleaning. Duplicate and incomplete records were removed during this process (Fig. 1).

### 2.3 Research methods

We queried the MeSH database to identify terms related to depression and mineral elements, as previously described. A comprehensive literature search was conducted in the selected WoS SCIE database (2007 to present), with the topic field searched using the "OR" operator for the selected terms, followed by the application of the "AND" operator. The data with complete records and references were downloaded, pre-processed, and imported into NoteExpress and CiteSpace for analysis.

We extracted data such as publication year, journal distribution, and citation frequency of the target literature. The distribution of literature sources and academic institutions, keyword co-occurrence, citation analysis, citation author co-occurrence, clustering, burst detection (strength, fronts, duration), and temporal changes (timelines, time zones) were analyzed.

Based on the quantitative analysis results and critical literature, we analyzed the current research status, discussing the role of mineral elements in depression, focusing on research frontiers and paradigm shifts. Finally, the results of the qualitative and quantitative analyses were integrated to draw systematic conclusions.

## 3 Results

### 3.1 Search results

The literature search in the SCIE database yielded 392,621 articles related to depression and 1,827,218 articles related to trace elements. By combining the search results using the "AND" operator, we obtained 16,838 articles. After removing non-English language literature and articles from unrelated categories. CiteSpace was used for data cleaning, which included removing duplicate records and verifying data integrity. The final dataset for analysis consisted of 12,648 articles.

### 3.2 General characteristics of the literature

Since 2007, the overall growth of the literature has been stable, with the most prominent years 2010 (an increase of 14.4 %), 2019 (9.2 %), and 2020 (8.7 %) (Appendix 1). The top 10 journals with the largest number of articles were Journal of Affective Disorders, Bipolar Disorders, PLOS ONE, Journal of Neuroscience, Progress in Neuro-Psychopharmacology and Biological Psychiatry, Scientific Reports, Neuropharmacology, Journal of Clinical Psychiatry, Behavioural Brain Research, and Neuroscience (Fig. 2a).

**Fig.2a:**
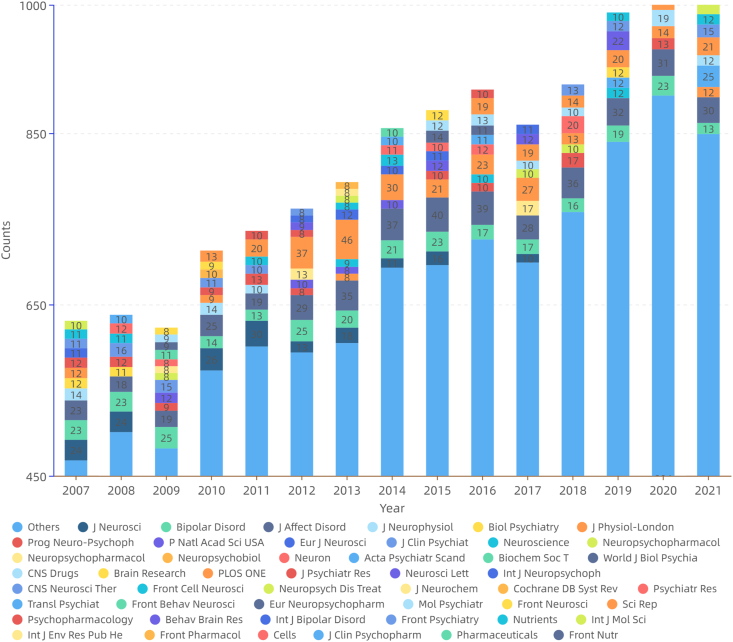
Annual journal distribution (general characteristics of literature).

The frequency of mineral elements in the keywords was as follows: lithium (1538 times), calcium (1236), iron (187), zinc (113), magnesium (71), potassium (58), selenium (13), sodium (10), cadmium (5), etc. The 14 elements were retrieved from the target literature collection managed by NoteExpress to obtain the approximate number of related literature as follows: lithium (4038), calcium (2113), zinc (1238), iron (771), magnesium (415), selenium (261), copper (191), iodine (103), manganese (97), chromium (54), lead (48), cobalt (42), fluorine (41), and molybdenum (20). These were plotted according to the annual publication volume (Fig. 2b).

**Fig.2b:**
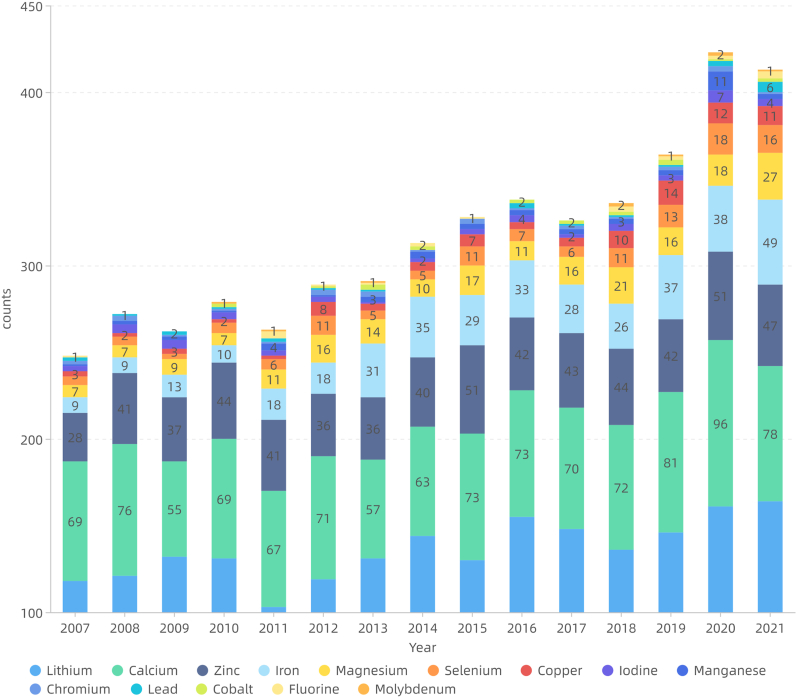
Annual distribution of mineral elements literature (general characteristics of literature).

The top 10 categories according to the number of articles were Neuroscience & Neurology, Neuroscience, Psychiatry, Pharmacy & Clinical Neurology, Biochemistry & Molecular Biology, Science & Technology-Other Topics, Multidisciplinary Sciences, Psychology, and Behavioural Sciences (Fig. 2c).

**Fig.2c:**
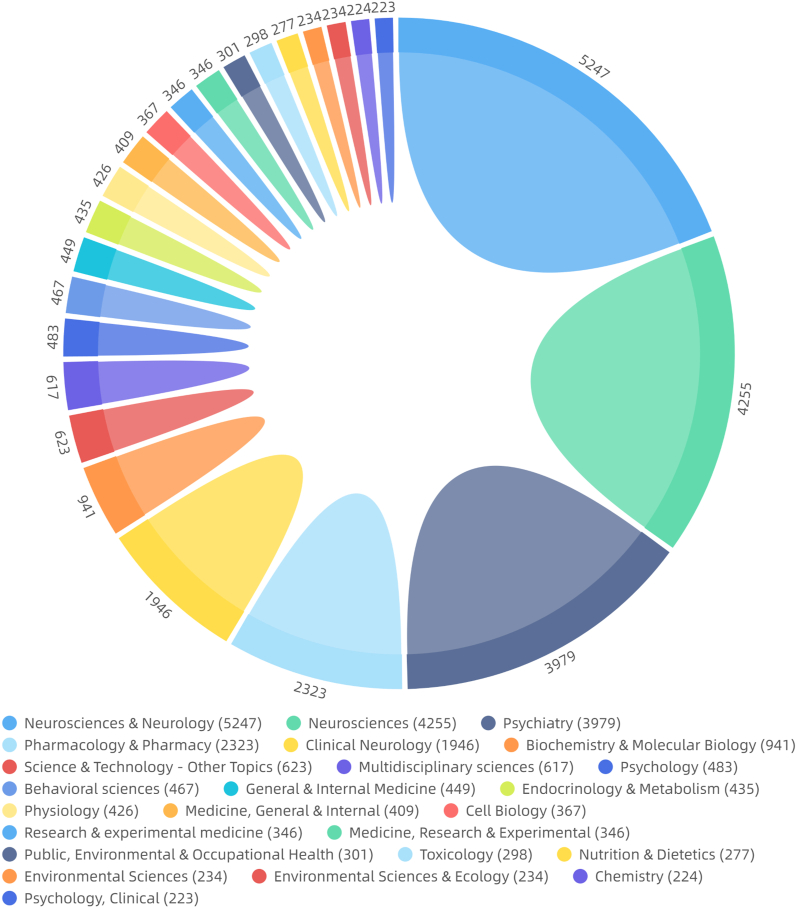
Discipline distribution of the literature (general characteristics of the literature).

The top 10 institutions with the highest number of publications were: University of Toronto, Harvard University, King's College London, University of Melbourne, University of Barcelona, Karolinska Institute, University of Pittsburgh, NIMH, and Massachusetts General Hospital (Fig. 2d).Institutions with a centrality value greater than or equal to 0.04 included King's College London, Chinese Academy of Sciences, University of Barcelona, Institut National de la Santé et de la Recherche Médicale, Heidelberg University, University of Oslo, Harvard University, University of Melbourne, National Institute of Mental Healt, University of California San Diego, and Brown University (Fig. 2e).

**Fig.2d:**
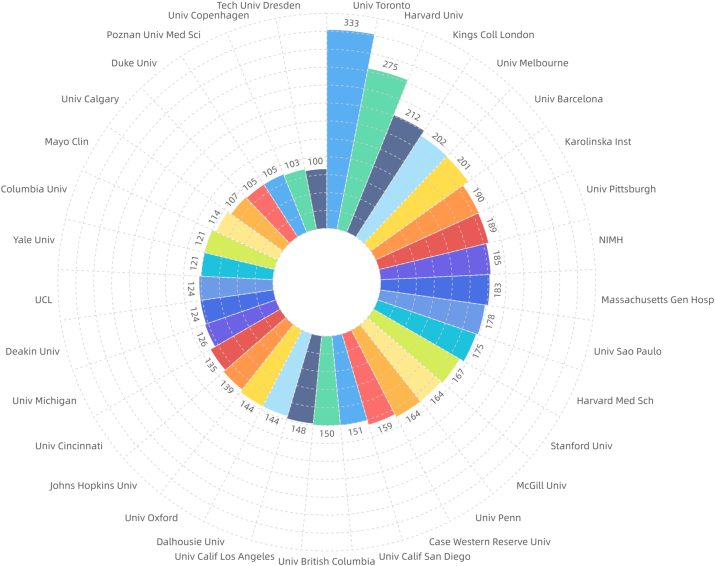
Distribution of major institutions with literature (general characteristics of literature).

**Fig.2e:**
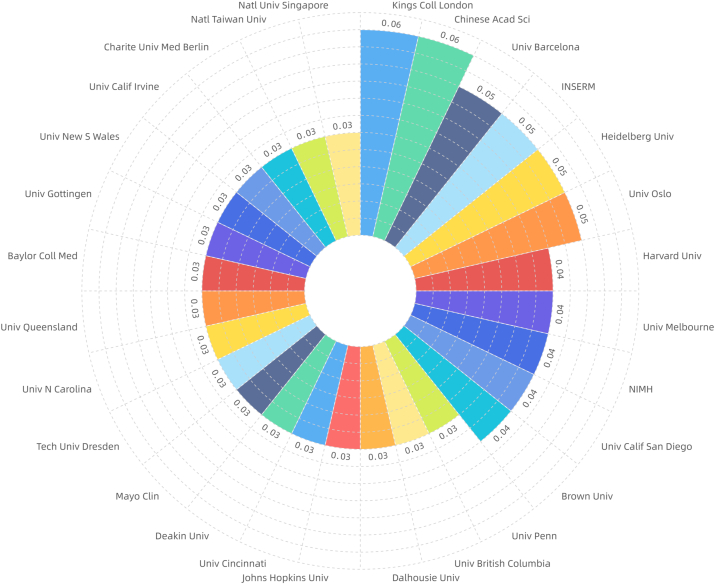
Distribution of institutions with higher centrality (general characteristics of literature).

The top 10 countries/regions contributing to the literature were the United States with 4619 (4619/18,642, 24.8 %, more than the sum of the next three countries), followed by China, United Kingdom, Canada, Germany, Italy, Brazil, Japan, Australia, and the Netherlands (Fig. 2f).

**Fig.2f:**
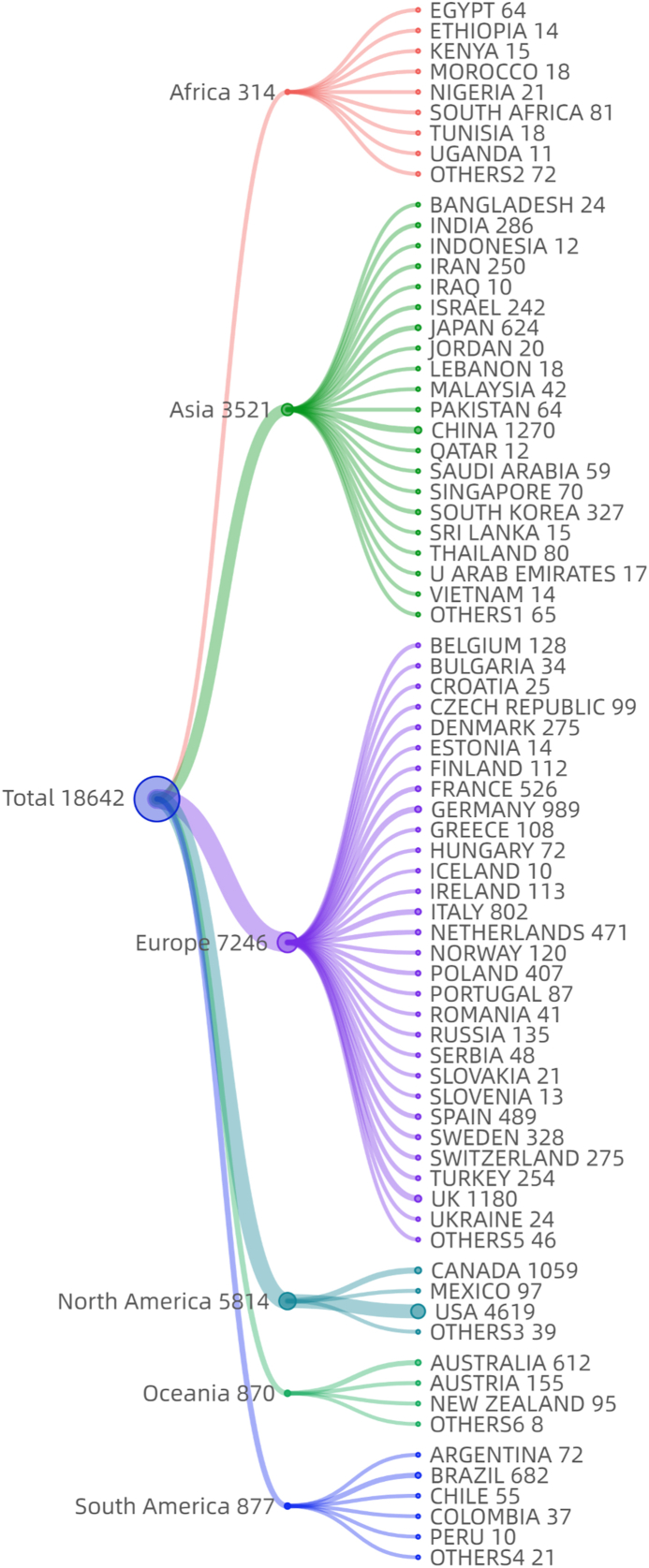
Country/Region distribution of literature (some articles are from more than one country/region due to international cooperation) (general characteristics of literature).

Regarding the most cited articles, 15 of the 20 most cited articles containing epidemiological studies on the most significant global burden of disease involving depression and mineral elements were from the assembly of the United States in the target literature. The top five articles are the global burden of disease reports published by *LANCET,* while the other five studies focus on the characteristics of disease in the world, the most populous country, or a specific population. In addition, there are four studies on genes, two on NMDA (N-Methyl-d-aspartate) and one on phytochemicals, animal models, synapses and ion channels for each (Appendix 2).

### 3.3 Cluster network co-occurrence analysis

#### 3.3.1 Keyword analysis

After CiteSpace ran, 54 pairs of synonyms were combined for formal operation analysis. The meaningful high frequency words identified were bipolar disorder, lithium, double-blind, brain, oxidative stress, synaptic plasticity, meta-analysis, Alzheimer's disease, prefrontal cortex, calcium, and calcium channel (Fig. 3a). Meaningful high-centrality words identified were Parkinson's disease, medial prefrontal cortex, calcium channel, forced swimming test, valproic acid, cortical spreading depression, hippocampal neurogenesis, spectrum disorder, apoptosis, augmentation, substance use, synaptic plasticity, meta-analysis, Alzheimer's disease, gene expression, and calcium (Fig. 3b). A heat map of the main high-frequency words indicated that bipolar disorder and depression had the most significant numbers and showed rapid growth. Lithium maintained long-term high popularity with slight fluctuations. Inflammation (emerging in 2013), oxidative stress, meta-analysis, neurotrophic factor, and prefrontal cortex showed pronounced growth trends, while expression, calcium, brain, mechanism, Alzheimer's disease also showed apparent growth (Fig. 3c). Frontier keywords included bipolar disorder, drinking water, systematic review, biomarker, inflammation, neuroinflammation, and gut microbiota.

**Fig. 3a:**
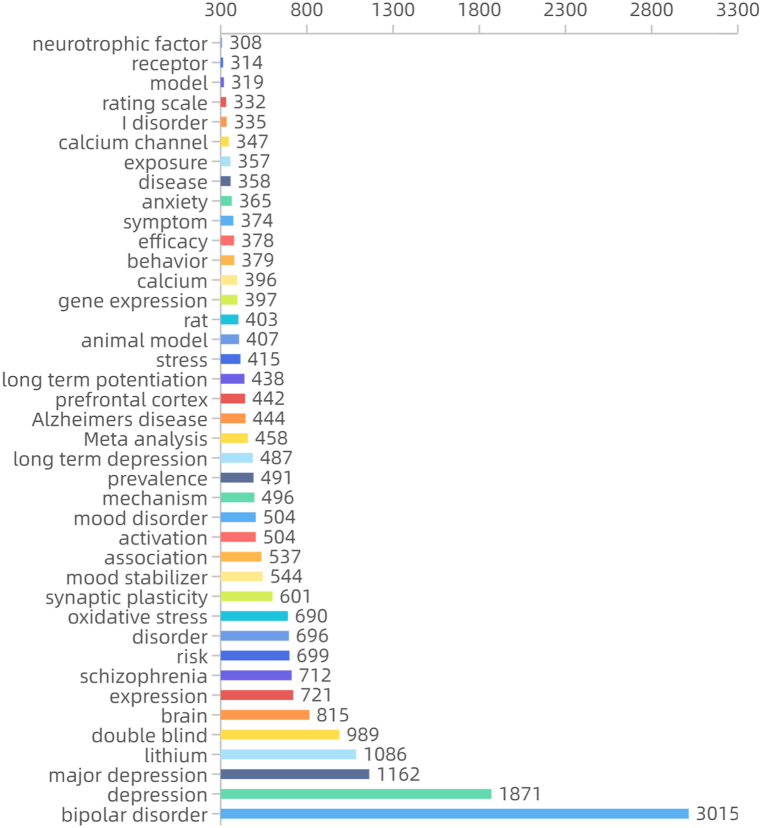
Keyword frequency (keyword analysis).

**Fig. 3b:**
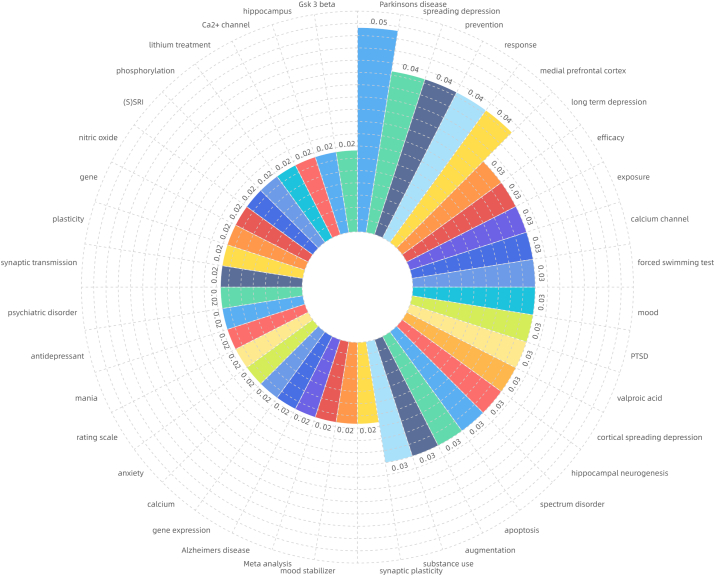
Keyword centrality (keyword analysis).

**Fig.3c:**
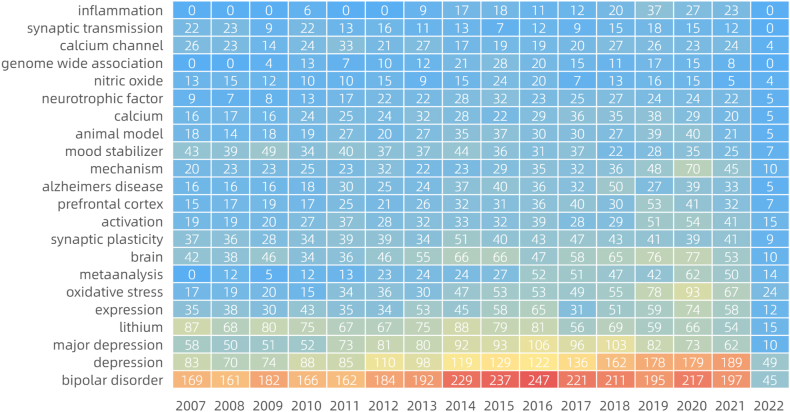
Heat map of the main keywords (keyword analysis).

#### 3.3.2 Citation analysis

**High-frequency citations:** Ranking the citation frequency, eight (40 %) of top 20 articles, which focused on BD and lithium, were from the United States, five were from the United Kingdom, and three were from Canada. Moreover, six were BD diagnosis and treatment guidelines, two were BD treatment reviews, and others included one book, four studies on lithium treatment of BD, two studies on lithium toxicity and side effects, four studies on BD genes, and one review on BD neural pathways (Table 1).

**Table 1:** Top 20 high-frequency citations.

No.	Author	Freq.	Title	Journal	Volume	Region
1	L.N. Yatham [37]	171	*The Canadian Network for Mood and Anxiety Treatments (CANMAT) and the International Society for Bipolar Disorders (ISBD) collaborative update of CANMAT guidelines for the management of patients with bipolar disorder: Update 2013*	*BIPOLAR DISORD*	2013,15(1)	CANADA
2	L.N. Yatham [38]	139	*Canadian Network for Mood and Anxiety Treatments (CANMAT) and International Society for Bipolar Disorders (ISBD) 2018 guidelines for the management of patients with bipolar disorder*	*BIPOLAR DISORD*	2018,20(2)	CANADA
3	APA [39]	116	Diagnostic and statistical manual of mental disorders, fifth edition	*APA PUBLISHING*	2013	USA
4	G.M. Goodwin [40]	116	*Evidence-based guidelines for treating bipolar disorder: Revised third edition recommendations from the British Association for Psychopharmacology*	*J PSYCHOPHARMACOL*	*2016,30(6)*	UK
5	R.F. McKnight [41]	110	*Lithium toxicity profile: A systematic review and meta-analysis*	*LANCET*	2012,379(9817)	UK
6	L.N. Yatham [42]	109	*Canadian Network for Mood and Anxiety Treatments (canmat) and International Society for Bipolar Disorders (ISBD) collaborative update of canmat guidelines for the management of patients with bipolar disorder: update 2009*	*BIPOLAR DISORD*	2009,11(3)	CANADA
7	A. Cipriani [43]	102	*Lithium in the prevention of suicide in mood disorders: Updated systematic review and meta-analysis*	*BMJ-BRIT MED J*	2013, 346	ITALY
8	I. Grande [44]	100	*Bipolar disorder*	*LANCET*	2016,387(10027)	SPAIN
9	L. Hou [45]	92	*Genetic variants associated with response to lithium treatment in bipolar disorder: A genome-wide association study*	*LANCET*	2016,387(10027)	USA
10	S. Ripke [46]	89	*Biological insights from 108 schizophrenia-associated genetic loci*	*NATURE*	2014,511(7510)	USA
11	J. Smoller [47]	88	*Identification of risk loci with shared effects on five major psychiatric disorders: A genome-wide analysis*	*LANCET*	*2013, 381(9875)*	USA
12	G.S. Sachs [48]	81	*Effectiveness of Adjunctive Antidepressant Treatment for Bipolar Depression*	*N ENGL J MED*	2007,356(17):	USA
13	J.R. Geddes [49]	80	*Lithium plus valproate combination therapy versus monotherapy for relapse prevention in bipolar I disorder (BALANCE): A randomized open-label trial*	*LANCET*	2010, 375(9712)	UK
14	M. Berk [50]	80	*Pathways underlying neuroprogression in bipolar disorder: Focus on inflammation, oxidative stress and neurotrophic factors*	*NEUROSCI BIOBEHAV R*	2011, 35(3)	AUSTRALIA
15	P. Sklar [51]	74	*Large-scale genome-wide association analysis of bipolar disorder identifies a new susceptibility locus near ODZ4*	*NAT GENET*	2011, 43(10)	USA
16	J.R. Geddes [52]	71	*Treatment of bipolar disorder*	*LANCET*	2013,381(9878)	UK
17	G.S. Malhi [53]	70	*Potential mechanisms of action of lithium in bipolar disorder. Current understanding*	*CNS DRUGS*	2013,27(2)	AUSTRALIA
18	F.K. Goodwin [54]	70	*Manic-Depressive Illness: Bipolar Disorders and Recurrent Depression, Second Edition*	*OXFORD UNIVERSITY PRESS*	2007	USA
19	G.M. Goodwin [55]	68	*Evidence-based guidelines for treating bipolar disorder: Revised second edition—recommendations from the British Association for Psychopharmacology*	*J PSYCHOPHARMACOL*	2009,23(4)	UK
20	M. Gitlin [56]	68	*Lithium side effects and toxicity: Prevalence and management strategies*	*INT J BIPOLAR DISORD*	2016,4(1)	USA

**Clustering timeline:** There is a noteable trend lithium response, magnet supplementation, treatment-resistant unipolar depression, and systematic review have rapidly gained attention from 2013 to the present. Other clusters have become weaker and more relaxed since 2008 (Fig. 4a).

**Fig.4a:**
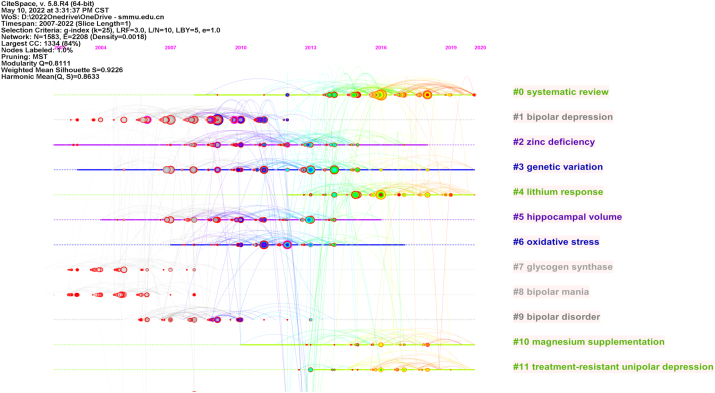
Citation clustering time-line (temporal distribution of citation clustering).

**Time zone:** High-frequency citations show two prominent dense areas centered around 2009 and 2013. The latter area is slightly less dense but recent, with notable articles by Goodwin GM(2016) [40], Grande I(2016) [44], Hou LP(2016) [45], and Yatham LN(2018) [38] being frontier and high centrality citations (Fig. 4b).

**Fig.4b:**
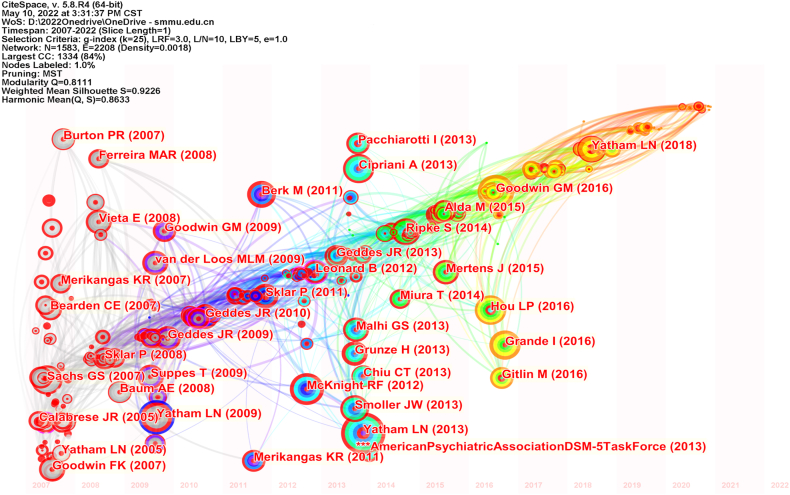
Citation time zone (temporal distribution of citation clustering).

**High-centrality citations:** Eight of the top 20 were from the United States (40 %), including 17 studies related to BD, 8 studies related to lithium, 4 oxidative stress, and others such as quetiapine, somatostatin, and pathological mechanism. (Appendix 3).

Quetiapine is more effective than other drugs in treating acute BD exacerbations [[57], [58], [59]], and the effect of ketamine is rapid and robust [27,60,61]. The pathological mechanism of BD is complex and difficult to clarify [62], involving monoamines, inflammation, cytokines, cortisol, neural factors, mitochondria, gut microbiota, etc. The theory of oxidative stress is more systematic and ideal [50,63]. Although changes in the nonspecific zone of the brain are noted, pinpointing pecise areas remains challenging [64].(Fig. 5a)

**Fig.5a:**
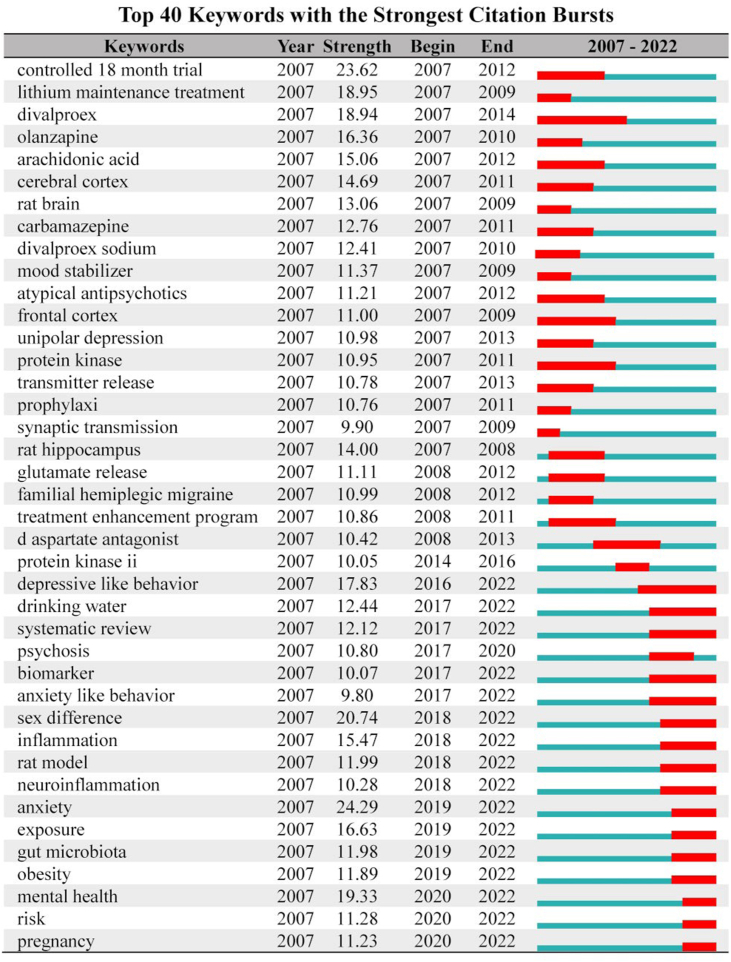
Top 40 burst keywords (burst analysis of keywords and citations).

Lithium can treat most acute episodes of BD episodes [65] and is effective in preventing suicide [43] and recurrence [49]. Decreased levels of lipid peroxidation after administration at the onset of BD suggest an antioxidant effect [66]. Multiple studies support the neuroprotective effects of these antioxidant mechanisms [37,53,65], including protecting the prefrontal cortex, hippocampus, and amygdala [53]. However, some patients do not respond to lithium treatment [67], possibly due to the expression of long non-coding RNAs AL157359.3 and AL157359.4 long non-coding RNAs [45]. There is also insufficient evidence supporting lithium efficacy in treating BD [59], with concern about its safety and the fact that only a few patients really benefit from it [62].

Depressive symptoms in GABA receptor mutant mice can be reversed by ketamine, and enhancing somatostatin-positive GABAergic interneurons can rapidly achieve antidepressant effects [68].

**Frontier citations:** Of the nine citations, five (55.6 %) were from the United States and 7 were BD-related studies (Fig. 5b, Appendix 4). Noteably, the *Evidence-based guidelines for treating BD, 3rd ed.* published by the British Psychopharmacological Association [40], is significant. Correct diagnosis for BD patients is challenging, but critically important for management [69], and familiarity with advances in pharmacology and psychology is essential for the diagnosis and treatment of BD [44]. The long noncoding RNA genes AL157359.3 and AL157359.4 are important regulators associated with individual differences in the response of BD to lithium therapy [45]. Moreover, 30 significant gene loci are associated with BD [70]. Canada recommends lithium, quetiapine, and sodium divalproex alone or in combination for the first-line treatment of acute mania in BD [38]. The number of prescriptions for lithium treatment for BD has recently declined due to concerns about the side effects [56]. Structural changes occur in the brains of patients with BD, but the findings are inconsistent [71]. Crosstalk between inflammatory pathways and neural circuits leads to avoidance and alertness, contributing to morbidity and non-response to antidepressant medication [72].

**Fig.5b:**
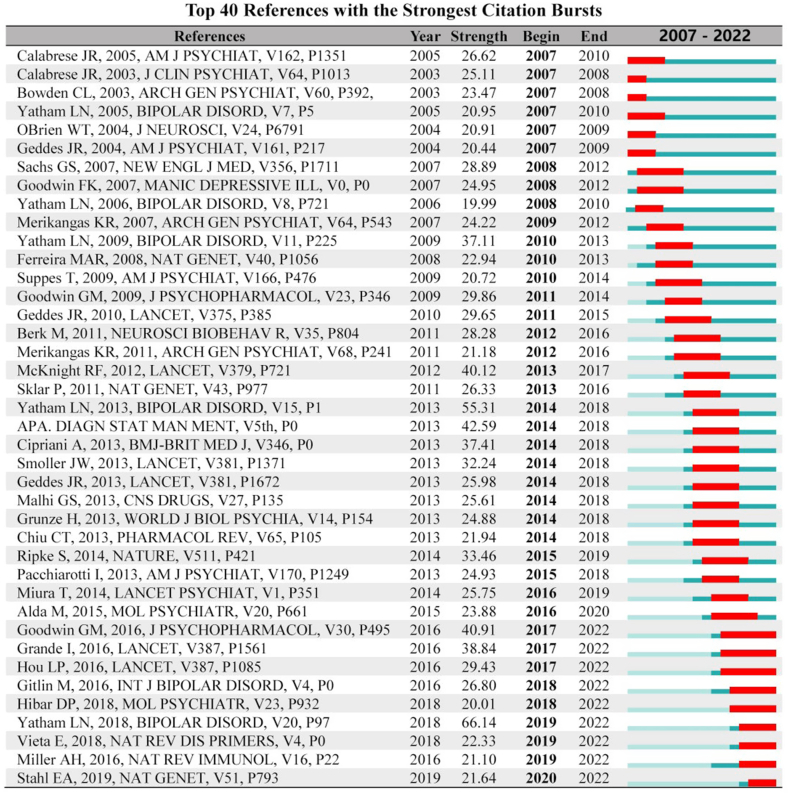
Top 40 burst citations (burst analysis of keywords and citations).

## 4 Discussion

### 4.1 General characteristics of the literature

The dramatic increase in the size of the literature can be attributed to several factors: (1) The global outbreak of the COVID-19 epidemic (Coronavirus Disease 2019) in 2019–2020 may have affected the incidence of depression [73], indirectly promoting the progress of related research; (2)The H1N1 epidemic outbreak in 2009 and the series of global adverse events in 2010, such as the earthquake in Haiti, the oil spill in the Gulf of Mexico, a volcanic eruption in Iceland, and the economic recession in the United States. Additionlly, a theoretical study on depression in 2010 made a significant discovery [25], leading to rapid progress [27] and a paradigm shift [26].

The main journals and disciplines in this field include neuroscience, nutrition, and pharmacy. Frontier disciplines are nutrition, medical informatics, and chemistry. Literature from the United States accounts for a high proportion of most-cited articles, high-frequency, high-centrality, and frontier citations. However, the top six influential academic institutions are from other countries/regions.

### 4.2 Research fronts and trends

**BD:** It is very prominent on the heat map, labelled as the largest keyword cluster remain active. The top 20 high-frequency citations, high-centrality citations, and frontier citations are all dominated by BD research. Among psychiatric disorders, BD has a more significant proportion of severe cases [74] and a higher number of YLD [74]. It is also one of the leading causes of disability among young people, with an increasing incidence in recent years [75]. BD is usually insidious and lacks specific biomarkers [44], complicating its diagnosis. Current features used for information include neuroimaging, peripheral measurements, and genetic findings are for information purposes only [76], such as microglia activation of microglia [77], BDNF (Brain-Derived Neurotrophic Factor) [78], proBDNF [79], and mineral element levels in vivo [16,80]. Epigenetic identification of BDs in response to lithium therapy reduces unnecessary toxic side effects [81]. Berk M(2011) [50] systematically reviewed the neuropathological process of BD and constructed a comprehensive model that included monoamines, inflammatory factors, steroids, neurotrophic factors, mitochondrial energy production, oxidative stress, neurogenesis, providing a theoretical basis for the diversified treatment of BD.

**Drinking water:** Being frontier keywords, many studies have reported that lithium levels in drinking water are inversely related to suicide, criminal, and cognitive impairment rates in different regions [[82], [83], [84], [85], [86], [87]]. Therefore, some experts recommend adding low-dose lithium to tap water as part of public health strategies [88].

**Lithium:** The heat map shows continuous high heat, especially from 2014 to 2016, with a slight setback recently. As the most crucial therapeutic drug for BD, lithium has been used clinically for nearly seven decades and remains the best mood stabilizer for relapsed/refractory depression and the most effective anti-suicide drug [43]. In recent years, physicians have become cautious in prescribing lithium due to its toxic side effects [89]. However, the concern seems superfluous due to the lack of significant adverse reaction [90]. Multiple problems can be solved by effective medication management, such as discontinuation before pregnancy and checking calcium concentration during treatment, and others [41,56]. Optimizing the medication regimen is recommended to benefit patients [56,89]. Additionally, lithium deposition in the liver and kidney can be reversed by selenium supplementation [91].

Mechanisms by which lithium treats BD include: (1) reducing dopamine and glutamate and increasing GABA neurotransmission; (2)regulating the second messenger to inhibit excessive excitatory transmission; (3) increasing BDNF and BCL-2 to protect nerves [53]; and (4) antioxidant effects [66].

**Inflammation and gut microbiota:** Frontier keywords include inflammation, neuroinflammation, and gut microbiota. Depression models show a decrease in antioxidant capacity, activation of the O&NS pathway, and destruction of ω-3 PUFAs, generating neoantigens that cause neurodegeneration following immune inflammation [92]. Increased TNFα and IFNγ production, activating indoleamine 2,3-dioxygenase (IDO), decrease in 5-HT synthesis, and inflammation collectively shaped the depressive phenotype [63]. Neuroinflammation induced in the hippocampus of rats persisted after stress, regardless of whether stress sensitive or resilient [93]. Treatment of BD by addressing oxidative stress and inflammation [50] improves depressive-like behaviours and mood disorders [[94], [95], [96], [97]].

The gut microbiota activates cytokines, O&NS, and IDO, thus affecting brain's changes of the 5-HT and NMDA receptors [63]. High-fat [98] or magnesium-deficient [99,100] diets, exposure to inhibiting stress [101], prenatal stress [102], electromagnetic exposure [103], and broad-spectrum antibiotic exposure [104] can alter gut bacteria, gastrointestinal inflammation, leading to increased depression and anxiety-like behaviours in mice.

**Paradigm shift:** Research on magnesium supplementation and treatment-resistant depression gained attention after 2013, as indicated by clustering citation clustering timelines. Concurrently, the term "brain" also showed increased prominence on heat maps, a trend that has persisted to the present.

The standardized definitions provided in the DSM-V (Diagnostic and Statistical Manual of Mental Disorders, Fifth Edition, 2013) have laid the groundwork for thriving research in this area. Szewczyk B (2008) [105] elucidated the antidepressant mechanism of zinc and magnesium within the theoretical framework of glutamate, BDNF, and GSK3 (glycogen synthase kinase 3). Perea G (2009) [24] demonstrated the involvement of glial cells in brain function, while Traynelis SF (2010) [25] found that glutamate receptors mediate fast excitatory synaptic transmission. Zarate CJ (2012) [27] repeatedly validated the rapid, powerful effects of a single dose of ketamine in treating BD patients. These findings catalyzed a shift in research paradigms [26], with receptor antagonists and magnesium being shown to affect synaptic connections and reverse neural changes after stress, thus opening new avenues for drug development [26]. The FDA's stance on ketamine has also evolved recently (2019) [106].

NMDA receptors characterized by voltage-dependent blockade of Mg^2+^ [107,108], are implicated in the pathological process of depression and exhibit antidepressant activity [109,110]. Intracellular magnesium declines with age, even when serum Mg^2+^ levels remain normal. Inflammation and oxidative stress, closely linked to aging, make the association between magnesium deficiency and aging-related neurological diseases plausible [111,112]. Extensive data from community sample data [113] and systematic reviews [114] suggest a correlation between magnesium intake and depressive symptoms. Although the causal direction remains to be determined, it does not affect the inclusion of magnesium supplementation in treatment strategies.

Magnesium supplementation has been shown to prevent and treat various diseases [115] and improve insomnia [109]. Magnesium deficiency is prevalent in chronic diseases [116,117], with risk factors including certain medications, alcohol consumption, and diets high in processed foods and refined grains [118]. Animal experiments, epidemiological studies, and clinical trials have confirmed the link between magnesium and depression [113,114,[119], [120], [121]]. Magnesium deficiency is manifested by excessive emotionality [122] and cognitive impairment, and chronic deficiency induces low-grade inflammation [112], which is the common pathological basis of many diseases [118], accompanied by an increase in inflammatory factors, acute phase proteins, and free radicals, which are often overlooked [123] due to ‘normal’ serum Mg^2+^ [111,124].

The magnesium level in cerebrospinal fluid is higher than in blood [125], where it blocks NMDA receptors and acts as a mood stabilizer, similar to ketamine. Magnesium positively affects depression associated with migraine [126,127], and cortical depression or glutamate neurotransmission abnormalities may be the mechanisms underlying the hypomagnesium-migraine association [128]. Long-term magnesium supplementation significantly reduces anxiety levels in rats [129] and normalize neuroinflammation and synaptic potentiation in mice [130]. Furthermore, symptoms improved after the correction for magnesium deficiency in patients with depression [131]. Thus, magnesium lactate to treat severe depression can improve efficacy [132].

### 4.3 Other elements

**Calcium:** Being a high-frequency and high-centrality keyword, ‘calcium channel’ in the heat map continues to be significant and “calcium” has grown significantly. The inflammatory activation of the NLRP3 endoplasmic reticulum BD (calcium dependent) is enhanced, reactive oxygen species accumulate [133], and intracellular calcium concentration is altered [134], which is the target point for verapamil and nimodipine antimania and mood stabilization [135]. Calcium supplementation and intake of dairy products can improve mood, but the correlation of dose with anxiety and stress perception is more complicated [19]. Synaptic plasticity is crucial for brain learning and memory and is dependent on cellular calcium influx [136]. Temporal cortical Ca^2+^ activity decreased during the depressive phase of mice but increased significantly during ketamine-induced mania [137].

**NMDA receptors** are voltage dependent and calcium-permeable [107,108], play an important role in the pathogenesis of depression [138,139], and are a critical factor in the immediate antidepressant effect of ketamine [140]. Calmodulin-expressing synapses can improve antidepressant efficacy [141], and inhibiting the expression of small-conductance calcium-activated potassium channel subtype-3 can reverse depression-related symptoms [142].

The neuropsychiatric disorder risk gene CACNA1C is closely related to depression, its abnormal expression disrupts Ca^2+^ homeostasis, leads to abnormal brain development, and increases anxiety [143]. CaV1.3-deficient mice do not exhibit anxiety or depression-like behaviours [144], and depression-like behaviors [144], and Cav1.2Δ33 plays a role in synaptic plasticity [145].

**Zinc and copper:** Zinc supplementation has a positive effect on depression [14,146], reduces depressive symptoms [147], improves the efficacy of antidepressants [148], and increases calcium and copper levels [149] when used as an adjunctive drug. BD zinc and copper levels fluctuate [[150], [151], [152]] due to mutual antagonism between the two trace elements [21]. However, both have antidepressant effects associated with the antagonization of NMDA receptors [153,154]. Changes in zinc levels and receptors may be the key to the successful treatment of depression with medication [155]. Furthermore, other mental disorders are also related to zinc and copper in the body [156].

Zinc, copper, iron, and selenium have antioxidant activities and play essential roles in the pathophysiology of oxidative stress that causes depression [[157], [158], [159]]. A meta-analysis of observational studies confirmed that dietary copper, selenium and manganese intakes are inversely associated with depression [18]. Trace concentrations of copper in patients with mood disorders were found to be reduced [16]. However, the heterogeneous results of some studies suggested that women with a high serum copper are more prone to depression [17,120], indicating uncertainty about the association. Copper is often deposited in the hippocampus and cortex, and excess can lead to decreased antioxidant capacity [160] and neurodegeneration [20]. High serum copper levels can be the pathological basis of major depress disorders [161]; a case of Wilson's disease copper overload manifested as depression was misdiagnosed and resulted in death [162]. NMDA receptor antagonists (Memantine) significantly inhibited serum copper levels while improving symptoms in depression models [163].

**Iron, selenium, and manganese:** Depression is caused by neurochemical changes involving neuronal plasticity and neurotransmitter (serotonin and GABA) metabolism [7]. Fe and Mn are involved in glutamatergic and GABAergic systems [10,12,15] and dopamine synthesis [164] and lead to extrapyramidal or cerebellar damage symptoms when deposits, with nonspecific psychiatric symptoms that are easily overlooked [165]. Fe, selenium, and manganese, like copper, are essential cofactors of enzymes involved in pathophysiological processes [15], such as SOD (superoxide dismutase) and glutathione peroxidase, playing a crucial role in antioxidant defense under normal conditions. Furthermore, they have been shown to have the potential to improve inflammation [13] and the severity of diseases [166].

Accumulated poisoning causes mitochondrial damage, produces lipid peroxides, and induces apoptosis. Selenium nanoparticles protected against oxidative damage in the rat hippocampus with a potent antioxidant, anti-inflammatory, and neuroprotective potential [167].

**Cobalt and chromium:** Trivalent chromium salts improve insulin efficiency, control blood sugar, and regulate mood disorders [168]. In addition, they have better glycaemic regulation effects in diabetic individuals with depression or binge eating disorders who have dopamine and serotonin disorders [169]. Cortical cadmium levels are lower in subjects with major depressive disorder, BD, and suicide completers [170], and chromium deficiency is rare. Serotonin metabolism depends on several nutritional cofactors, such as pyridoxine and mineral elements (chromium, zinc) [171]. Chromium picolinate reverses depressive and anxious behaviour in rats, possibly associated with increased 5-HT and decreased corticosterone levels [172]. Chromium antidepressant activity has also been suggested to be atypical, independent of serotonin receptors and transporters, and not the result of changes in the adrenergic system [173].

People with partial eclipse and digestive dysfunction often lack vitamin B_12_, causing depression and mental and emotional disorders [174]. CoCl_2_ acts as a synaptic activity blocker for treating frontal cortex-related chronic head pain and depression [175], and pretreatment of the neostriatum with CoCl_2_ reduces the incidence and duration of vigilance, defensive immobility, and escape of explosiveness escape [176]. CoCl_2_ induces the expression of microRNA-146a, improves the efficacy of mesenchymal stem cells (hUCB-MSCs) in the treatment of inflammatory diseases, and promotes the expression of anti-inflammatory mediators (PGE_2_) and cytokines (TNF-α and IFN-γ) to play a part in Ref. [177].

**Molybdenum:** Ammonium tetrathiomolybdate (TTM) can remove copper ions from copper thiolate clusters (such as SOD1), and played a therapeutic role in a mouse model of familial amyotrophic lateral sclerosis (ALS) caused by the SOD1 mutation [23], decreasing spinal copper levels and reducing lipid peroxidation. TTM can reverse the evasion of cancer cells from cisplatin-induced death through copper transporters (such as ATPase copper transporting β) [22]. Molybdenum cofactor (Moco) deficiency is a rare metabolic disorder that leads to sulfite accumulation in the brain [178] with neurological dysfunction and seizures.

**Iodine:** Urinary iodine is lower in depressed adolescents [179]. In areas where iodized salt is not widely used, early use can boost children's cognitive development [180], while later use improves their intelligence [181]. Postnatal thyroid hormone deficiency disrupts the structure and function of the cortical and limbic area, leading to neurological and psychiatric disorders, which l-thyroxine can improve [182]. The deiodinase enzyme regulates thyroid hormone levels, and downregulation of the calretinin gene alters GABAergic transmission in type 2 deiodinase knockout mice [183].

### 4.4 Mineral element toxicity

**Lead:** Overexposure triggers a series of psychiatric symptoms (anxiety and depression) that can be fatal in severe cases [184]. Lead-mediated receptors (ryanodine receptors, RyRs) up-regulate calcium levels, trigger the calcium-dependent signaling pathway (CaMKIIα/CREB) inhibition, activate the Erk/Bcl2 apoptosis pathway, and finally lead to neurodegeneration [185]. It can cause behavioral and cognitive deficits in childhood, with long-term effects such as cognitive decline in old age [186], and altered protein expression [187]. Blood lead levels were significantly associated with depression and the gut microbiota were involved [188]. Lactobacillus and Bifidobacterium decreased after exposure to lead, and 5-HT levels in blood and striatum decreased [189]. Although numerous studies have shown that exposure to lead is associated with neurological damage, a meta-analysis did not demonstrate a significant association between lead and multiple neurological markers, including mood changes [187].

**Fluorine:** It is a common serious pollutant [190]. After exposure of rats, investigators found: (1)inhibition of catalase, transaminase, and acetylcholinesterase (AChE) in the striatum of offspring [191]; (2)alteration in the composition and structure of the gut microbiota [192]; (3)activation of microglia, release pro-inflammatory cytokines [193]; and (4) changes in the structure of granules [194]. Collectively, these changes induce neuroinflammation, affecting neurodevelopment, secondary behavioural disorders, and intellectual impairment. Additionally, increased expression of BDNF4 and 5-HT1A [195], neural excitation-inhibition imbalance [196], cognitive deficits and anxiety [195], and behavioural abnormalities and depression [196] were observed in mouse.

Changes in the mitochondrion cause learning and memory impairment [194], childhood ADHD, and emotional irregularities [197]. Epidemiological studies have found similar results [198], and meta-analyses also support the impact of high fluoride exposure on IQ [199]. Antioxidant-rich hesperidin restores acetylcholinesterase activity and antioxidant stress parameters and alleviates neurobehavioral disorders induced by fluoride exposure [200].

**Cobalt:** Recent reports have linked cobalt poisoning with the growing use of metal joint implants [201]. Exposure symptoms include cognitive deficits, depression [202], memory loss, and mood disturbances [203]. A study (n = 247) found that brain hypometabolism was statistically significant in all subjects, 30 % statistically significant, even though only half had elevated blood/urine cobalt [204], suggesting that neurotoxicity precedes measurable elevations [201]. In contrast, 53 implant cases with high serum cobalt reported only increased subjective symptoms, without apparent neurotoxicity, possibly due to insufficient exposure time [205].

**Others:** After high manganese exposure in rats, 5-HT decreased in the cortex, hippocampus, and striatum [206,207]. Patients with chronic migraine [208] or Parkinson's disease [209] have localized iron deposits in the brain, and the iron chelator deferiprone improves symptoms [209]. Symptom onset is delayed after exposure to a methyl iodide, leading to late development of cognitive and psychiatric disorders, without an antidote available [210]. Lithium poisoning occurs at concentrations as low as 10 mg/L, with a risk of death of 20 mg/L when used in high doses [211].

## 5 Conclusions

The rapid growth in publications over time indicates that two factors may have contributed to the increase in research in this field. The first is the indirect impact of global adverse events on depression prevalence; The second is the facilitating effect of significant discoveries and influential publications in depression research.

The articles are published in relatively dispersed journals but are relatively concentrated in specific disciplines, authors' countries/regions, and influential academic institutions. Mainstream journal categories include psychiatric-neuroscience, pharmacology, and general journals. Main disciplines include neuroscience, biochemistry-molecular biology, interdisciplinary, and behavioral sciences. While publications from the US dominate, the 6 most influential institutions are actually from other countries/regions.

BD remains a research frontier, consistently depicted in warm hues on heatmaps and slightly less intense in recent years. The significant role of lithium salts in its treatment is still firmly established. Concerns about the side effects of these salts may be overstated. Enhancing medication management and optimizing administration regimens can mitigate side effects while maximizing therapeutic benefits for BD patients. Research has indicated a correlation between higher lithium levels in drinking water and lower suicide rates in those regions. Therefore, introducing low-dose lithium into tap water should be considered a potential public health policy option. The pathogenesis of depression is closely linked with inflammatory responses and alterations in gut microbiota. The regulation of these processes may emerge as novel therapeutic targets for depression.

A paradigm shift in depression research began in 2013, primarily driven by several influential papers and the standardized definitions provided by DSM-V (2013). These studies highlighted the immense potential of magnesium supplementation in treating treatment-resistant depression, exerting significant pharmacological effects through its influence on glutamate, BDNF, and GSK3. It should be noted that a magnesium deficiency led to an increase in neural inflammation. Furthermore, a correlation has been established between the levels of magnesium in the body and depressive symptoms. In both animal models of depression and patients, magnesium supplementation has been shown to alleviate anxiety and depressive symptoms, thus demonstrating its promise as a novel approach to treating treatment-resistant depression.

In addition to lithium and magnesium, depression has been found to have a significant correlation with a variety of trace elements. For example, calcium ions play a critical role in the regulation of synaptic plasticity; Zinc has been shown to alleviate depressive symptoms through its antagonistic effect on NMDA receptors. Despite their antagonistic relationship, both zinc and copper have been found to exhibit antidepressant properties. Elements such as copper, selenium, and manganese participate in oxidative stress processes, and their levels are inversely correlated with the incidence of depression. Chromium salts enhance metabolism, while cobalt mitigates depression by suppressing inflammatory factors. An iodine deficiency disrupts thyroid hormone synthesis, leading to cognitive decline; Meanwhile, a deficiency in molybdenum results in sulfide accumulation in the brain.

Notably, overexposure to various mineral elements, including copper, lead, fluorides, cobalt, manganese, iron, and iodides, can induce neuroinflammation, oxidative stress, neurotransmitter abnormalities, apoptosis, and other mechanisms, leading to varying degrees and types of nervous system damage and mental disorders.

In summary, lithium currently has definitive antidepressant efficacy among mineral elements. Magnesium, calcium, zinc, and manganese have varying antidepressant effects through NMDA receptor interactions and other biochemical actions. Current research frontiers include BD, inflammatory responses, and the gut microbiota, with several key mineral elements shaping current and future directions in this field.

## 6 Limitations

Due to CiteSpace's requirement for literature records with references to conduct valuable citation analysis, the SCIE database (2007-present) meets this requirement and represents the academic mainstream literature. Therefore, the conclusions drawn from the study of 12,648 articles of SCIE are highly reliable and robust. The limitation of noisy data in the 12,648 articles is addressed by the characteristics of CiteSpace, where irrelevant literature forms small, marginalized clusters that ultimately do not affect the results and conclusions.

The algorithm of CiteSpace is still being continuously improved and upgraded. Future versions of CiteSpace may yield slightly different results when analyzing the same dataset.

## Funding statement

This work was supported by the 10.13039/501100001809National Natural Science Foundation of China (81573150), and Military Key Discipline Construction Projects of China (HL21JD1206), and Project of Naval Medical University (2022MS002).

## Data availability statement

Data supporting the findings of this study are openly available in Mendeley Data at https://doi.org/10.17632/v4nkkc99nn.1 (https://data.mendeley.com/datasets/v4nkkc99nn/1), reference number v4nkkc99nn.1, and are available from the corresponding author on reasonable request.

## Ethics statement

Not applicable.

## CRediT authorship contribution statement

**Biao Gao:** Writing – original draft, Software, Methodology, Investigation, Formal analysis, Conceptualization. **Chenqi Li:** Writing – original draft, Software, Methodology, Investigation, Formal analysis, Conceptualization. **Yicui Qu:** Writing – original draft, Software, Methodology, Investigation, Formal analysis, Conceptualization. **Mengyu Cai:** Writing – original draft, Data curation. **Qicheng Zhou:** Software, Investigation. **Yinyin Zhang:** Supervision, Resources. **Hongtao Lu:** Visualization, Software. **Yuxiao Tang:** Writing – review & editing, Data curation. **Hongxia Li:** Writing – review & editing, Supervision, Resources, Funding acquisition, Conceptualization. **Hui Shen:** Writing – review & editing, Supervision, Resources, Funding acquisition, Conceptualization.

## Declaration of competing interest

The authors declare the following financial interests/personal relationships which may be considered as potential competing interests:Shen Hui reports financial support was provided by 10.13039/501100001809National Natural Science Foundation of China. All other authors, they declare that they have no known competing financial interests or personal relationships that could have appeared to influence the work reported in this paper.

## Figures and Tables

**Figure:**
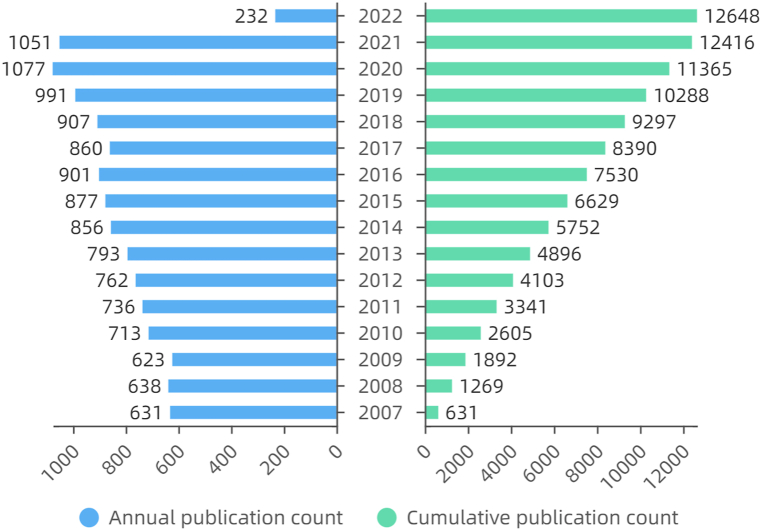


**Table:** 

No.	Author	Freq.	Title	Journal	Volume	Region
1	T. Vos et al.	4424	*Years lived with disability (YLD) for 1160 sequelae of 289 diseases and injuries 1990-2010*	*LANCET*	2012, 380(9859)	USA
2	T. Vos et al.	3707	*Global, regional, and national incidence, prevalence, and years lived with disability for 310 diseases and injuries, 1990-2015*	*LANCET*	2016, 388(10053)	USA
3	T. Vos et al.	3437	*Global, regional, and national incidence, prevalence, and years lived with disability for 301 acute and chronic diseases and injuries in 188 countries*	*LANCET*	2015, 386(9995)	USA
4	S. James et al.	3289	*Global, regional, and national incidence, prevalence, and years lived with disability for 354 diseases and injuries for 195 countries and territories*	*LANCET*	2018, 392(10159)	USA
5	T. Vos et al.	2667	*Global, regional, and national incidence, prevalence, and years lived with disability for 328 diseases and injuries for 195 countries, 1990-2016*	*LANCET*	2017, 390(10100)	USA
6	S. Traynelis et al.	2188	*Glutamate Receptor Ion Channels: Structure, Regulation, and Function*	*PHARMACOL REV*	2010, 62(3)	USA
7	J. Smoller,et al.	1872	*Identification of risk loci with shared effects on five major psychiatric disorders: a genome-wide analysis*	*LANCET*	2013, 381(9875)	USA
8	N. Li et al.	1756	*mTOR-Dependent Synapse Formation Underlies the Rapid Antidepressant Effects of NMDA Antagonists*	*SCIENCE*	2010, 329(5994)	USA
9	C. Murray et al.	1601	*The State of US Health, 1990–2010 Burden of Diseases, Injuries, and Risk Factors*	*JAMA*	2013, 310(6)	USA
10	L. Duley	1494	*The Global Impact of Pre-eclampsia and Eclampsia*	*SEMIN PERINATOL*	2009, 33(3)	ENGLAND
11	E. Nestler et al.	1274	*Animal models of neuropsychiatric disorders*	*NAT NEUROSCI*	2010, 13(10)	USA
12	G. Yang et al.	1258	*Rapid health transition in China, 1990–2010: findings from the Global Burden of Disease Study 2010*	*LANCET*	2013, 381(9882)	USA
13	J. Xiong et al.	1256	*Impact of COVID-19 pandemic on mental health in the general population: A systematic review*	*J AFFECT DISORDERS*	2020, 277	CANADA
14	S. Walker et al.	1138	*Child development in developing countries 2 - Child development: risk factors for adverse outcomes in developing countries*	*LANCET*	2007, 369(9556)	JAMAICA
15	S. Ripke et al.	1031	*Genome-wide association analysis identifies 13 new risk loci for schizophrenia*	*NAT GENET*	2013, 45(10)	USA
16	G. Perea et al.	1006	*Tripartite synapses: astrocytes process and control synaptic information*	*TRENDS NEUROSCI*	2009, 32(8)	SPAIN
17	B. Nilius,et al.	996	*Transient receptor potential cation channels in disease*	*PHYSIOL REV*	2007, 87(1)	BELGIUM
18	P. Sklar et al.	947	*Large-scale genome-wide association analysis of bipolar disorder identifies a new susceptibility locus near ODZ4*	*NAT GENET*	2011, 43(10)	USA
19	M. Ferreira et al.	875	*Collaborative genome-wide association analysis supports a role for ANK3 and CACNA1C in bipolar disorder*	*NAT GENET*	2008, 40(9)	USA
20	P. Anand et al.	822	*Biological activities of curcumin and its analogues (Congeners) made by man and Mother Nature*	*BIOCHEM PHARMACOL*	2008, 76(11)	USA

**Table:** 

No.	Author	Cent.	Title	Journal	Volume	Region
1	S.L. McElroy et al.	0.14	*A double-blind, placebo-controlled study of quetiapine and paroxetine as monotherapy in adults with bipolar depression (EMBOLDEN II)*	*J CLIN PSYCHIAT*	2010, 71(2)	USA
2	C.A. Zarate Jr et al.	0.13	*Replication of ketamine's antidepressant efficacy in bipolar depression: a randomized controlled add-on trial*	*BIOL PSYCHIAT*	2012, 71(11)	USA
3	B. Leonard et al.	0.11	*Mechanistic explanations how cell-mediated immune activation, inflammation and oxidative and nitrosative stress pathways and their sequels and concomitants play a role in the pathophysiology of unipolar depression*	*NEUROSCI BIOBEHAV R*	2012, 36(2)	IRELAND
4	L.N. Yatham et al.	0.10	*Canadian Network for Mood and Anxiety Treatments (CANMAT) and International Society for Bipolar Disorders (ISBD) collaborative update of CANMAT guidelines for the management of patients with bipolar disorder: update 2013*	*BIPOLAR DISORD*	2013, 15(1)	CANADA
5	J. Mertens et al.	0.10	*Differential responses to lithium in hyperexcitable neurons from patients with bipolar disorder*	*NATURE*	2015, 527(7576)	CHINA
6	M.E. Thase et al.	0.10	*Efficacy of Quetiapine Monotherapy in Bipolar I and II Depression: A Double-blind, Placebo-controlled Study (The BOLDER II Study)*	*J CLIN PSYCHOPHARM*	2006, 26(6)	USA
7	M. Berk et al.	0.09	*Pathways underlying neuroprogression in bipolar disorder: focus on inflammation, oxidative stress and neurotrophic factors*	*NEUROSCI BIOBEHAV R*	2011, 35(3)	AUSTRALIA
8	G.S. Malhi et al.	0.09	*Therapeutic Mechanisms of Lithium in Bipolar Disorder: Recent Advances and Current Understanding*	*CNS DRUGS*	2016, 30(10)	AUSTRALIA
9	L. Hou et al.	0.08	*Genetic variants associated with response to lithium treatment in bipolar disorder: a genome-wide association study*	*LANCET*	2016,387(10027)	USA
10	R.T. de Sousa et al.	0.08	*Oxidative stress in early stage Bipolar Disorder and the association with response to lithium*	*J PSYCHIATR RES*	2014, 50	BRAZIL
11	A.H. Young et al.	0.07	*A double-blind, placebo-controlled study of quetiapine and lithium monotherapy in adults in the acute phase of bipolar depression (EMBOLDEN I)*	*J CLIN PSYCHIAT*	2010, 71(2)	CANADA
12	M.M. Sidor et al.	0.07	*Antidepressants for the acute treatment of bipolar depression: a systematic review and meta-analysis*	*J CLIN PSYCHIAT*	2011, 72(2)	USA
13	P. Zanos et al.	0.07	*NMDAR inhibition-independent antidepressant actions of ketamine metabolites*	*NATURE*	2016, 533(7604)	USA
14	N.C. Brown et al.	0.07	*An updated meta-analysis of oxidative stress markers in bipolar disorder*	*PSYCHIAT RES*	2014, 218(1–2)	CANADA
15	I. Pacchiarotti et al.	0.06	*The International Society for Bipolar Disorders (ISBD) task force report on antidepressant use in bipolar disorders*	*AM J PSYCHIAT*	2013, 170(11)	SPAIN
16	M.J. Kempton et al.	0.06	*Meta-analysis, database, and meta-regression of 98 structural imaging studies in bipolar disorders*	*ARCH GEN PSYCHIAT*	2008, 65(9)	ENGLAND
17	T. Fuchs et al.	0.06	*Disinhibition of somatostatin-positive GABAergic interneurons results in an anxiolytic and antidepressant-like brain state*	*MOL PSYCHIATR*	2017, 22(6)	USA
18	A. Cipriani et al.	0.05	*Lithium in the prevention of suicide in mood disorders: updated systematic review and meta-analysis*	*BMJ*	2013, 346	ITALY
19	J.R. Geddes et al.	0.05	*Lithium plus valproate combination therapy versus monotherapy for relapse prevention in bipolar I disorder (BALANCE): a randomised open-label trial*	*LANCET*	2010, 375(9712)	UK
20	G.S. Malhi et al.	0.05	*Potential mechanisms of action of lithium in bipolar disorder. Current understanding*	*CNS DRUGS*	2013, 27(2)	AUSTRALIA

**Table:** 

No.	Author	Strength	Title	Journal	Volume	Region
1	G.M. Goodwin et al.	40.91	*Evidence-based guidelines for treating bipolar disorder: Revised third edition recommendations from the British Association for Psychopharmacology*	*J Psychopharmacol.*	*2*016,30(6)	UK
2	I. Grande et al.	38.84	*Bipolar disorder*	*LANCET*	2016,387(10027)	SPAIN
3	L. Hou et al.	29.43	*Genetic variants associated with response to lithium treatment in bipolar disorder: a genome-wide association study*	*LANCET*	2016,387(10027)	USA
4	M. Gitlin	26.8	*Lithium side effects and toxicity: prevalence and management strategies*	*Int J Bipolar Disord*	2016,4(1):27	USA
5	D.P. Hibar et al.	20.01	*Cortical abnormalities in bipolar disorder: an MRI analysis of 6503 individuals from the ENIGMA Bipolar Disorder Working Group*	*Mol Psychiatry*	2018,23(4)	USA
6	L.N. Yatham et al.	66.14	*Canadian Network for Mood and Anxiety Treatments (CANMAT) and International Society for Bipolar Disorders (ISBD) 2018 guidelines for the management of patients with bipolar disorder*	*Bipolar Disord*	2018,20(2)	CANADA
7	E. Vieta et al.	22.33	*Bipolar disorders*	*Nat Rev Dis Primers*	2018,4	SPAIN
8	A.H. Miller et al.	21.10	*The role of inflammation in depression: from evolutionary imperative to modern treatment target*	*Nat Rev Immunol*	2016,16(1):	USA
9	E.A. Stahl et al.	21.64	*Genome-wide association study identifies 30 loci associated with bipolar disorder*	*Nat Genet*	2019,51(5)	USA

**Table:** 

(MeSH)	Medical subject headings
(BD)	Bipolar disorder
(YLD	Lived with disability
(WOS)	Web of science
(SCIE)	Science citation index expanded
(GABA)	Gamma-aminobutyric acid
(COVID-19)	Coronavirus disease 2019
(BDNF)	Brain-derived neurotrophic factor
(IDO)	Indoleamine 2,3-dioxygenase
(NMDA)	N-methyl-d-aspartate
(DSM-V)	Diagnostic and statistical manual of mental disorders, fifth edition
(GSK3)	Glycogen synthase kinase 3
(FDA)	Food and drug administration
(SOD)	Superoxide dismutase
(ADHD)	Attention-deficit and hyperactivity disorder
(IQ)	Intelligence quotient
